# Comparative study between efficacy of topical tofacitinib 2% ointment versus topical clobetasol propionate 0.05% ointment in hand eczema at a tertiary hospital in central India

**DOI:** 10.12688/f1000research.147725.1

**Published:** 2024-04-23

**Authors:** Drishti Bhatt, Dr Adarshlata Singh

**Affiliations:** 1 Dermatology, Venereology and Leprosy, Datta Meghe Institute of Higher Education and Research, Wardha, 442001, India

**Keywords:** Hand eczema, Topical Tofacitinib, Clobetasol Propionate, Dermatology, Randomized controlled trial, Hand Eczema Severity Index (HECSI)

## Abstract

**Background:**

Hand eczema is a prevalent dermatological condition that significantly impacts the quality of life of affected individuals. Topical corticosteroids, such as Clobetasol Propionate, are commonly employed for management, but concerns regarding long-term use and potential side effects necessitate exploration of alternative treatments. This study protocol outlines a randomized controlled trial comparing the efficacy of topical Tofacitinib 2% ointment with Clobetasol Propionate 0.05% ointment in managing hand eczema.

**Methods:**

The study will be a single-blinded, parallel-group, randomized controlled trial conducted at the Dermatology outpatient department of Acharya Vinoba Bhave Rural Hospital Sawangi Meghe, Wardha, from March 2023 to September 2025. Eligible participants diagnosed with hand eczema will be randomized into two groups: Group 1 will receive Clobetasol Propionate 0.05% ointment, and Group 2 will receive Tofacitinib 2% ointment. The primary outcome measure is the change from baseline in the Hand Eczema Severity Index (HECSI) score, assessed at two weeks and four weeks. Statistical analysis will utilize appropriate parametric and non-parametric tests, with a p-value <0.05 considered significant.

**Expected Outcome:**

The study is anticipated to provide insights into the comparative efficacy and safety of Tofacitinib and Clobetasol Propionate in managing hand eczema. A favorable outcome for Tofacitinib could suggest its potential as an alternative or adjunctive therapy, offering a more targeted approach with fewer side effects. The findings may contribute to optimized treatment strategies for hand eczema, improving patient outcomes and guiding future research in dermatological therapeutics.

## Introduction

Hand eczema is a common dermatological condition characterized by inflammation of the skin on the hands, leading to symptoms such as erythema, vesicles, fissures, and pruritus. ^1^ It significantly impacts the quality of life of affected individuals, causing discomfort and functional impairment. ^2^ The management of hand eczema often involves topical corticosteroids, with Clobetasol Propionate being a commonly prescribed agent. ^3^ However, concerns regarding the long-term use of potent corticosteroids, potential side effects, and the need for alternative treatments have prompted exploration into novel therapeutic options.

One emerging candidate for managing inflammatory skin conditions is Tofacitinib, a Janus kinase (JAK) inhibitor that modulates cytokine signaling and has demonstrated efficacy in conditions like psoriasis. ^4^ Tofacitinib’s mechanism of action, targeting critical pathways involved in skin inflammation, makes it a promising candidate for hand eczema management.

While studies have explored the efficacy of topical corticosteroids like Clobetasol Propionate in hand eczema, there needs to be more comparative research evaluating the potential advantages of Tofacitinib over traditional treatments. This study seeks to address this gap by conducting a randomized controlled trial comparing the efficacy and safety of topical Tofacitinib 2% ointment with topical Clobetasol Propionate 0.05% ointment in managing hand eczema.

### Aim

To study the efficiency of topically applied tofacitinib 2% ointment in managing hand eczema.

### Objectives

1. To evaluate the efficacy of topical tofacitinib 2% ointment in hand eczema.
2. To evaluate the efficacy of topically applied clobetasol propionate 0.05% ointment for hand eczema.
3. To compare the effect of topically applied tofacitinib 2% ointment and topically applied clobetasol propionate 0.05% ointment in hand eczema.
4. To study the side effects of topical clobetasol propionate 0.05% ointment and topical Tofacitinib 2% ointment.

## Protocol

### Study design

This research will be conducted as a prospective, randomized, single-blinded, parallel-group, controlled trial to compare the efficacy of topical tofacitinib 2% ointment versus topical clobetasol propionate 0.05% (10-15 g depending on the area affected over 4 weeks Reagent number: 25122-46-7) ointment in the management of hand eczema.

### Participants

Patients diagnosed with hand eczema attending the outpatient department of Dermatology (DVL) at AVBRH Sawangi Meghe, Wardha, from March 2023 to September 2025, will be eligible for participation.

### Inclusion criteria

1. Patients providing written consent before the initiation of any treatment.
2. Clinically diagnosed cases of hand eczema of all grades as per the Hand Eczema Severity Index (HECSI) score.
3. Age above 18 years.
4. No prior treatment for hand eczema in the past 1 month.

### Exclusion criteria

1. Patients with an active infection at the local site.
2. Patients with leukopenia or anemia.
3. Pregnant or nursing women.
4. Patients on any treatment that may influence the outcomes of the drugs under study.

### Allocation

Patients meeting the inclusion criteria and providing written consent will be assigned to either Group 1 or Group 2 using computer-generated randomization. To minimize bias, age, and gender cross-matching between the groups will be implemented.

### Interventions

**Group 1 (Control): Topical Clobetasol Propionate 0.05% Ointment.** Patients in this group will receive topical Clobetasol Propionate 0.05% ointment as their treatment modality for hand eczema. They will be instructed to apply the ointment twice daily over the affected areas. The treatment duration will extend for 4 weeks.

**Group 2 (Experimental): Topical Tofacitinib 2% Ointment.** Patients in this group will receive topical Tofacitinib 2% ointment as their treatment modality for hand eczema. Similar to Group 1, they will be instructed to apply the ointment twice daily over the affected areas. The treatment duration will also be 4 weeks.

**Application instructions:** Patients in both groups will be educated on properly applying the assigned ointments. They will be instructed to wash their hands before applying a thin layer of the ointment evenly on the affected areas, avoiding contact with eyes and mucous membranes. Patients will be advised not to bandage or cover the treated areas unless directed by the investigator. Additionally, they will be instructed to avoid excessive sun exposure and use sunscreen on treated areas.

**Follow-up visits:** Participants from both groups will be scheduled for followup visits at 2 weeks and 4 weeks after the initiation of treatment. During these visits, clinical photographs will be taken with the patient’s informed consent, and the Hand Eczema Severity Index (HECSI) ^5^ score will be recorded to assess the efficacy and side effects of the respective interventions.

**Monitoring:** The study will be monitored to ensure adherence to the assigned interventions, proper data collection, and participant safety. Adverse events, if any, will be documented and reported. Regular communication with participants will be maintained to address any concerns or queries they may have during the study.

**Adjustments:** Any deviations from the study protocol will be documented, and appropriate measures will be taken to address them. Adjustments will be made in consultation with the Institutional Ethics Committee if necessary.

**Blinding:** The study will be single-blinded, with the participants unaware of their assigned treatment group. This helps minimize bias in reporting outcomes.

**Code break:** In a medical emergency or if unblinding becomes necessary for clinical management, a code break procedure will be in place, ensuring that the treating physician can access information about the assigned intervention while keeping the participant blinded.

**Adherence monitoring:** Participants will be asked to return unused medication at each followup visit to assess adherence. Additionally, self-reported adherence will be recorded during followup assessments. These interventions and allocation procedures aim to systematically evaluate the comparative efficacy and side effects of topical Clobetasol Propionate 0.05% ointment and topical Tofacitinib 2% ointment in managing hand eczema.

### Enrollment

1. **Recruitment:** The recruitment process will commence at the Dermatology outpatient department (DVL) of AVBRH Sawangi Meghe, Wardha, from March 2023. A designated study coordinator will identify potential participants based on clinical diagnosis and eligibility criteria. Eligible patients will be provided with detailed information about the study, and the purpose, risks, and benefits will be explained.
2. **Informed consent:** Written and signed informed consent will be obtained from all eligible patients willing to participate in the study. The consent form will include information about the nature of the study, potential risks and benefits, confidentiality assurances, and the voluntary nature of participation.
3. **Screening and inclusion/exclusion criteria:** Eligible participants will undergo a screening process to confirm their clinical diagnosis and ensure they meet the inclusion criteria while excluding those who meet any exclusion criteria. The study coordinator will collect relevant medical history and conduct a thorough examination to confirm the diagnosis of hand eczema.
4. **Baseline assessments:** Baseline assessments will include the collection of demographic information, medical history, and baseline Hand Eczema Severity Index (HECSI) scores. Clinical photographs of affected areas will be taken during followup visits for documentation and comparison.
5. **Randomization and allocation:** After obtaining informed consent and confirming eligibility, participants will be assigned to either Group 1 (Clobetasol Propionate) or Group 2 (Tofacitinib) through computer-generated randomization. Age and gender cross-matching will be performed to ensure balanced groups.
6. **Assignment of interventions:** Participants will be provided with the assigned treatment (topical Clobetasol Propionate 0.05% ointment or topical Tofacitinib 2% ointment) along with detailed instructions for application.
7. **Follow-up appointments:** Participants will be scheduled for followup visits at 2 weeks and 4 weeks after the initiation of treatment. The study coordinator will communicate the importance of attending all followup appointments and the need for adherence to the assigned treatment.
8. **Monitoring adherence:** Adherence to the assigned intervention will be monitored through self-reporting, return of unused medications, and documentation during followup visits.
9. **Data collection:** Data collection will include clinical assessments, HECSI score recording, and the capture of clinical photographs during each followup visit.
10. **Adverse event reporting:** Participants will be encouraged to report any adverse events or concerns during the study. Adverse events will be documented, assessed for severity, and reported to the Institutional Ethics Committee as per regulatory requirements.
11. **End of study:** The study will conclude in September 2025, and participants will be thanked for participating. Final data analysis and interpretation will be conducted, and the study findings will be disseminated through publications and presentations.

### Sample size

Effect of topical tofacitinib for hand eczema = 81.7%

Effect of topical steroid for hand eczema = 68%

Clinically significant at (δ) = 25%

$$N={\left(1.64+0.84\right)}^{2}*\left(0.817\right)\;\left(1–0.817\right)+0.68\;\left(1-0.68\right)/{\left(0.25\right)}^{2}=36$$

Sample size = 36

Total sample size = 72 (36 per group)

### Statistical analysis

The statistical analysis for this comparative study between topical Tofacitinib 2% ointment and topical Clobetasol Propionate 0.05% ointment in managing hand eczema will encompass a comprehensive approach utilizing appropriate parametric and non-parametric tests. The primary focus will be on the change from the baseline of the Hand Eczema Severity Index (HECSI) score. In the initial stage, descriptive statistics will be employed to summarize the baseline characteristics of participants in both treatment groups. Continuous variables will be presented as mean and standard deviation (or median and interquartile range), while categorical variables will be expressed as frequencies with percentages.

The comparative analysis will be conducted using an independent t-test or Mann-Whitney U test for the primary outcome—change from baseline in HECSI score. The choice between tests will depend on the data distribution, and statistical significance will be determined with a threshold of p<0.05. After the primary analysis, subgroup analyses may be undertaken to explore potential variations in treatment response based on age, gender, and baseline severity. Safety and adverse events will be summarized, and the incidence rates between the two treatment groups will be compared using Chi-square or Fisher’s exact test.

The handling of missing data will be addressed, potentially utilizing multiple imputations to enhance the robustness of the results. Sensitivity analyses may be performed to assess the impact of outliers or influential data points on the overall findings.

Exploratory analyses will investigate potential relationships between treatment response and additional factors, such as patient demographics or specific characteristics of hand eczema. Statistical software such as R Studio Version 4.3.1 will execute these analyses.

## Discussion

Hand eczema is a prevalent dermatological condition that substantially burdens affected individuals, impacting their daily lives and often requiring long-term management. ^6^ The choice of topical corticosteroids, such as Clobetasol Propionate, is a conventional approach, but concerns about prolonged use and potential side effects necessitate exploring alternative therapeutic options. ^7^ This study protocol outlines the design of a randomized controlled trial comparing the efficacy of topical Tofacitinib 2% ointment with topical Clobetasol Propionate 0.05% ointment in managing hand eczema.

The rationale for exploring Tofacitinib lies in its mechanism of action as a Janus kinase (JAK) inhibitor, which modulates intracellular signaling pathways involved in the inflammatory response. ^8^ While Tofacitinib has shown promise in conditions like psoriasis, ^9^ its efficacy and safety in hand eczema remain to be elucidated. The comparison with Clobetasol Propionate, a well-established treatment, will provide valuable insights into the potential advantages of Tofacitinib in terms of efficacy and side effect profiles.

The study’s methodology, including the use of a single-blinded randomized controlled trial design, rigorous inclusion and exclusion criteria, and standardized outcome measures, enhances the internal validity of the findings. Including a placebo group could have provided additional insights, but ethical considerations and the necessity of active treatment for hand eczema guided the decision to compare two active treatments.

The choice of the Hand Eczema Severity Index (HECSI) score as the primary outcome measure aligns with its established use in assessing the severity of hand eczema. ^5^ This comprehensive scoring system considers multiple clinical signs and provides a quantifiable measure for evaluating treatment efficacy.

One potential limitation of this study is its hospital-based nature, which may limit the generalizability of the findings to the broader population. Additionally, the study’s relatively short duration may not capture long-term effects or relapse patterns. However, the study’s strengths, including the robust study design, systematic randomization, and rigorous statistical analyses, contribute to its credibility and potential impact on clinical practice.

### Ethical considerations

1. The Institutional Ethics Committee of Datta Meghe Institute of Higher Education and Research (DU) has approved the study protocol date 21/03/23. Before commencing the study, we will obtain written informed consent from all participants, providing them with a comprehensive explanation of the study’s objectives. We will prioritize the interviewee’s privacy and comfort during the interview process. Reference Number: DMIHER (DU)/IEC/2024/70.

### Dissemination

This study protocol will be published in an indexed journal.

### Study status

The study has not yet started; we will start after the published study protocol.

## Data Availability

No data are associated with this article. Zenodo: SPIRIT checklist for ‘Comparative Study Between Efficacy of Topical Tofacitinib 2% Ointment vs Topical Clobetasol Propionate 0.05% Ointment in Hand Eczema in tertiary hospital in central India”, https://doi.org/10.5281/zenodo.10935094. ^10^ Data are available under the terms of the Creative Commons Attribution 4.0 International license (CC-BY 4.0).
