# Supplementary material for: Comparative study between efficacy of topical tofacitinib 2% ointment versus topical clobetasol propionate 0.05% ointment in hand eczema at a tertiary hospital in central India
Source: F1000Res. 2024 Apr 23;13:317. [Version 1] doi: 10.12688/f1000research.147725.1 (PMC11760495; doi:10.12688/f1000research.147725.1)
Supplement: Peer review history

# Reviewer report: Reviewer response for version 1

**DOI:** 10.5256/f1000research.161953.r361112

**Published:** 2025-01-24

**Authors:** Snehal Shelke

**Affiliations:** 1 CPR Government Hospital, Kolhapur, Maharashtra, India

Comparative clinical trial between topical tofacitinib Vs clobetasol propionate. The reduction in symptoms/signs as well as improvement in QOL can be included as viewed by the primary investigator and as stated by the patient, given that the primary objective is to look for a reduction in the HECSI score only. A follow up at 6 months after completion of initial 4 weeks would be interesting to look for any relapses as only topical therapy is being given and relapse rates of topical JAK STAT inhibitors for eczema is not well known. Given that among topical treatment options, steroids are the mainstay as of now for eczema treatment, it is interesting to think of topically applied JAK STAT inhibitor as an option to decrease the well known long term side effects of topical steroids. It would be of interest to know if, after the 4 week period, the patients are started on any concomitant oral therapy and if so then which mode of topical responds better if used along with oral treatment.

Is the study design appropriate for the research question?

Yes

Is the rationale for, and objectives of, the study clearly described?

Yes

Are sufficient details of the methods provided to allow replication by others?

Yes

Are the datasets clearly presented in a useable and accessible format?

Yes

Reviewer Expertise:

My area of expertise is allergic dermatitis like atopy and eczema and infectious diseases like  onychomycosis.

I confirm that I have read this submission and believe that I have an appropriate level of expertise to confirm that it is of an acceptable scientific standard.

# Reviewer report: Reviewer response for version 1

**DOI:** 10.5256/f1000research.161953.r290372

**Published:** 2024-06-26

**Authors:** Francisco José Navarro-Triviño

**Affiliations:** 1 Universitario San Cecilio, Granada, Spain

Comparative clinical trial between topical tofacitinib Vs clobetasol propionate. The main objective is the reduction of HECSI, although I think it should add the change in symptoms such as itching and pain using the NRS scale. It would be interesting if at the end of the first phase of the trial, patients who do not improve with clobetasol propionate receive topical tofacitinib.

 The follow-up time seems short to me, 4 weeks may be an early cut-off to assess the short-term response to the treatment.

Is the study design appropriate for the research question?

Yes

Is the rationale for, and objectives of, the study clearly described?

Yes

Are sufficient details of the methods provided to allow replication by others?

Yes

Are the datasets clearly presented in a useable and accessible format?

Yes

Reviewer Expertise:

My area of ​​expertise is the world of immune-mediated diseases, including eczematous-type inflammatory pathology.

I confirm that I have read this submission and believe that I have an appropriate level of expertise to confirm that it is of an acceptable scientific standard.
